# Nano-Derived Therapeutic Formulations with Curcumin in Inflammation-Related Diseases

**DOI:** 10.1155/2021/3149223

**Published:** 2021-09-15

**Authors:** Cristina Quispe, Natália Cruz-Martins, Maria Letizia Manca, Maria Manconi, Oksana Sytar, Nataliia Hudz, Mariia Shanaida, Manoj Kumar, Yasaman Taheri, Miquel Martorell, Javad Sharifi-Rad, Gianfranco Pintus, William C. Cho

**Affiliations:** ^1^Facultad de Ciencias de la Salud, Universidad Arturo Prat, Avda. Arturo Prat 2120, Iquique 1110939, Chile; ^2^Faculty of Medicine, University of Porto, Alameda Prof. Hernani Monteiro, Porto, Portugal; ^3^Institute for Research and Innovation in Health (i3S), University of Porto, Porto, Portugal; ^4^Institute of Research and Advanced Training in Health Sciences and Technologies (CESPU), Rua Central de Gandra, 1317, 4585-116 Gandra, PRD, Portugal; ^5^Department of Scienze della Vita e dell'Ambiente, Drug Science Division, University of Cagliari, 09124 Cagliari, Italy; ^6^Department of Plant Physiology, Faculty of Agrobiology and Food Resources, Slovak University of Agriculture, 94976 Nitra, Slovakia; ^7^Department of Plant Biology, Educational and Scientific Center “Institute of Biology and Medicine”, Kiev National University of Taras Shevchenko, Volodymyrska, 64, 01033 Kyiv, Ukraine; ^8^Department of Drug Technology and Biopharmaceutics, Danylo Halytsky Lviv National Medical University, Pekarska 69, Lviv, Ukraine; ^9^Department of Pharmacognosy and Medical Botany, I. Horbachevsky Ternopil National Medical University, Voli 1, Ternopil, Ukraine; ^10^Chemical and Biochemical Processing Division, ICAR–Central Institute for Research on Cotton Technology, Mumbai 400019, India; ^11^Phytochemistry Research Center, Shahid Beheshti University of Medical Sciences, Tehran, Iran; ^12^Department of Nutrition and Dietetics, Faculty of Pharmacy, and Centre for Healthy Living, University of Concepción, 4070386 Concepcion, Chile; ^13^Unidad de Desarrollo Tecnológico, Universidad de Concepción UDT, Concepcion 4070386, Chile; ^14^Department of Medical Laboratory Sciences, College of Health Sciences and Sharjah Institute for Medical Research, University of Sharjah, 22272 Sharjah, UAE; ^15^Department of Biomedical Sciences, University of Sassari, 07100 Sassari, Italy; ^16^Department of Clinical Oncology, Queen Elizabeth Hospital, Kowloon, Hong Kong

## Abstract

Due to its vast therapeutic potential, the plant-derived polyphenol curcumin is utilized in an ever-growing number of health-related applications. Here, we report the extraction methodologies, therapeutic properties, advantages and disadvantages linked to curcumin employment, and the new strategies addressed to improve its effectiveness by employing advanced nanocarriers. The emerging nanotechnology applications used to enhance CUR bioavailability and its targeted delivery in specific pathological conditions are collected and discussed. In particular, new aspects concerning the main strategic nanocarriers employed for treating inflammation and oxidative stress-related diseases are reported and discussed, with specific emphasis on those topically employed in conditions such as wounds, arthritis, or psoriasis and others used in pathologies such as bowel (colitis), neurodegenerative (Alzheimer's or dementia), cardiovascular (atherosclerosis), and lung (asthma and chronic obstructive pulmonary disease) diseases. A brief overview of the relevant clinical trials is also included. We believe the review can provide the readers with an overview of the nanostrategies currently employed to improve CUR therapeutic applications in the highlighted pathological conditions.

## 1. Introduction

The incidence of chronic diseases, including cardiovascular (CV), cerebrovascular (CeV), neurodegenerative, metabolic, pulmonary, autoimmune, endocrine, and osteoarticular, is alarming growing worldwide [1–6]. In this regard, the widely recognized crosstalk between inflammation, oxidative stress, and excessive proinflammatory cytokine production results in one of the main triggering factors in promoting the onset and progression of the aforementioned chronic conditions [7–9].

Increasing evidence indicates a critical interplay between oxidative stress and inflammation in disease pathogenesis. Reactive oxygen species (ROS) released from inflammatory cells lead to oxidative stress, which is widely recognized as the direct link between the inflammatory process and disease onset and progression [10–12]. Indeed, both ROS and reactive nitrogen species (RNS) boost cell signaling pathways linked to increased proinflammatory gene expression, despite inflammation is regarded as a self-defense response of the human body to hazards, including injuries or allergens [4]. In this regard, synthetic medicines have been widely used for controlling and suppressing inflammation, although they are often associated with a plethora of undesirable side effects. On the other hand, natural antioxidants and anti-inflammatory (AIF) agents have shown instead the ability to achieve the necessary pharmacological impact with the lowest side effects compared to commonly used synthetic drugs [4, 13–15].

Naturally occurred bioactive products have been a source of new therapeutic medications for decades [15–19]. In this regard, curcumin (1,7-bis(4-hydroxy-3-methoxyphenyl)-1,6-heptadiene-3,5-dione; CUR) is a bioactive molecule isolated from *Curcuma longa* L. rhizomes with a plethora of therapeutic applications [20]. Synonymously known as diferuloylmethane, CUR is the major polyphenolic compound of *Curcuma* spp. [10, 21] with the ability to target various cell signaling pathways and modulate a wide range of biological activities [22]. Being CUR a natural and virtual nontoxic compound is the object of an intense number of investigations. Among others, CUR's AIF effects result from its ability to interact with multiple molecules and modulate the activity of several intracellular signaling pathways. Indeed, CUR has been reported to interact with the cellular redox status and modulates the activity of several protein kinases. CUR can downregulate inflammatory reaction-related transcription factors, cytokines, and enzymes that promote inflammation, besides to be able to activate the cellular apoptotic process via receptor- and mitochondrial-mediated pathways in a caspase-dependent fashion [23, 24]. Nonetheless, despite its vast therapeutic potentialities, as most of the natural bioactive compounds [25], CUR suffer of low bioavailability, partially due to its poor stability and solubility in the digestive tract, which ultimately restricts its therapeutic uses. Some catalyst compound-based approaches have been used to improve CUR bioavailability, including novel liquid and solid oral delivery systems, which have been tested to counteract both low CUR absorption and faster excretion from the human body [26]. In addition, nanotechnology-based CUR formulations have also been designed and tested for treating various diseases [27–29]. In this context, several types of nanoparticles, including micelles, polymeric nanoparticles, liposomes, solid lipids, nanogels, dendrimers, niosomes, silvers, and cyclodextrins, have been found suitable for CUR loading or encapsulation to improve its effectiveness as therapeutic agent in various diseases [30, 31].

First, extraction methodologies, therapeutic properties, and advantages and disadvantages of CUR employment are briefly addressed in this review. Then, new emerging nanoformulations and nanodelivery systems aimed at improving CUR efficacy against selected oxidative stress- and inflammatory-associated diseases such as wounds, arthritis, psoriasis, colitis, Alzheimer atherosclerosis, asthma, and chronic obstructive pulmonary diseases are collected, analyzed, and discussed.

## 2. Origin and General Bioactivity of Curcumin

CUR is the dominant polyphenol found in turmeric (*C. longa*) rhizomes and less often in other *Curcuma* species [1, 10, 32, 33]. Turmeric is a perennial herbaceous plant widely grown and cultivated in tropical and subtropical regions of South Western and Southern Asia [32–34]. Turmeric rhizomes contain essential oil (4.2–14%), fatty oil (8.76–12.7%), CUR (up to 5%), and other phytoconstituents [2]. The major turmeric essential oil components are ɑ-turmerone (42.6%), *β*-turmerone (16.0%), and ar-turmerone (12.9%), which are responsible for its potent antioxidant, antifungal, and antibacterial effects [35, 36].

CUR was formerly isolated from turmeric about two centuries ago, whereas its structure was elucidated around one century ago (in 1910) [3]. CUR is considered the most important turmeric secondary metabolite [4, 37], although two other dominant curcuminoids are also present, namely, demethoxycurcumin and bis-demethoxycurcumin [37]. Column chromatography has been the majorly used technique for CUR separation from the curcuminoid mixture [33]. CUR content in crude curcuminoids' powder is around 76.8%, whereas in recrystallized powder, its purity can be as higher as 99.45% [38].

**Figure 1:**
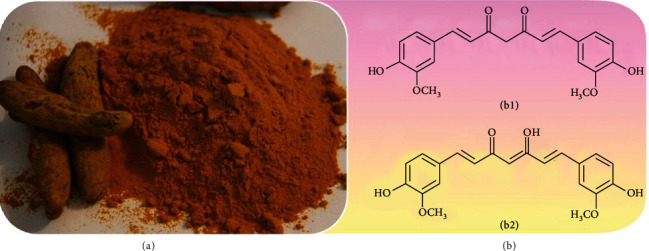
Turmeric (a) as a source of CUR; tautomeric forms of CUR (b): keto-form (B1) and enol-form (B2).

CUR has a bright yellow-orange color and exists in two tautomeric forms associated with different properties and activities. The keto-enol form is present in neutral or acid solutions, and the enol-form in alkalis solutions (Figure 1). CUR exists in its enolic form in ethanol or other organic solvents, while its keto form predominately presents in water [39]. Over time, and with the increasing number of studies performed, researchers have recognized the methoxy groups on CUR phenyl rings responsible for its therapeutic effects [10].

**Figure 2:**
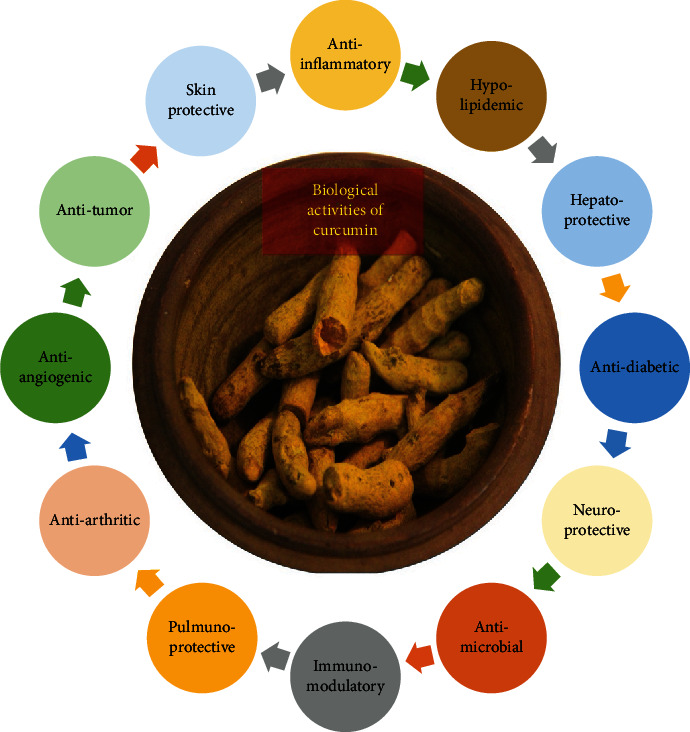
Biological activities of curcumin.

Turmeric is a key ingredient in both traditional Chinese medicine and Indian holistic systems. China and India population have been using CUR for centuries to treat infectious, skin disorders, depression, and stress [34, 40]. Turmeric is also used as a spice ingredient in curry and a coloring food additive compound, E100 [41]. It is also widely used as an herbal supplement for food and beverage flavoring and coloring [10]. Based on the recent findings suggesting monotargeted therapies less effective than multitargeted ones, turmeric can be considered the ideal “Spice for Life” [3]. Indeed, numerous pharmacological effects have been ascribed to CUR, the most abundant turmeric bioactive compound, including antioxidant, AIF, antitumor, antimicrobial, hypolipidemic, antidiabetic, neuroprotective, and hepatoprotective activities (Figure 2) [1, 37, 40]. But, worth noting is that most CUR's health benefits are mainly ascribed to its AIF and antioxidant properties [3].

CUR oral administration has no toxic effect on animals, and human studies have shown that its intake (up to 6 g/day) caused no toxicity even though prolonged for several weeks [24]. Moreover, based on the Food and Drug Administration (FDA), CUR consumption at a dose as high as 8 g per day is considered safe [10]. Thus, given all these properties, CUR can be regarded as an excellent potential candidate for nutraceuticals and pharmaceuticals formulation. Nonetheless, CUR presents some usage limitations, such as low bioavailability and low water solubility, which markedly limits its uses and therapeutic efficacy [10]. In this sense, as referred above, various strategies have been developed to overcome such obstacles in order to obtain effective CUR formulations [40, 42], with a progressively higher bioavailability and improved applicability [43]. For instance, other natural compounds, such as piperine, well-known for its CUR bioavailability enhancers action, have also been included CUR formulation. In this regard, data obtained so far reveal that piperine inclusion can raise CUR bioavailability by 2000% [10]. Also, more recently, chemistry computations have highlighted that CUR solubility improves in natural deep eutectic solvents [44].

## 3. General Overview of Curcumin Inflammation Modulatory Properties

*C. longa* has a very long history of use in the Indian system of medicine, known as Ayurveda, and in Chinese traditional medicine [45, 46]. Epidemiological observations suggest that turmeric consumption exerts protective effects in humans, decreasing the risk of several diseases, especially those associated with chronic inflammation and oxidative stress [34]. These properties are considered paramount in people's modern society modus vivendi characterized by lack of sleep, exaggerate consumption of junk food, alcohol, cigarette smoke, environmental pollutants, and stress (chemical, physical, mechanical, or psychological) which are the most common triggering factors of several inflammatory chronic diseases. Indeed, these factors have been now recognized as the main ones responsible for excessive free radical production and activation of proinflammatory factors, such as TNF and NF-*κ*B [47], which boost neurodegenerative, cardiovascular, pulmonary, metabolic, and autoimmune diseases [3].

Different synthetic drugs have been tested and successfully used to treat these diseases; however, many of them are linked to numerous side effects, which often reduce their effectiveness or are even responsible for therapy failure. [48, 49]. In this light, CUR has appeared as a promising bioactive capable of preventing or controlling oxidative stress and chronic inflammation, either used as a supplement or adjuvant in different pathologies to promote the beneficial effects or reduce the toxicity of synthetic drugs [50–54].

CUR antioxidant and AIF activities are regarded as the key components underpinning this naturally occurring compound's plethora of health benefits [1, 10, 55]. Indeed, CUR's antioxidant activity has been tightly connected to its ability to trigger several intracellular signaling pathways and modulates multiple cell functions. Various preclinical (*in vitro* and *in vivo*) and clinical studies have shown that CUR is a beneficial compound for inflammatory disease treatment and prevention [23, 56]. CUR can indeed promote its antioxidant activity by scavenging ROS and RNS, as well as by regulating the activity of key antioxidant enzyme systems such as superoxide dismutase, catalase, and glutathione reductase [5, 43]. Furthermore, CUR lipophilic properties also make it an excellent peroxyl radicals' absorber with chain-breaking antioxidant properties [10, 11]. On the other hand, this polyphenol is a well-documented AIF agent [23, 57], being able to counteract not only oxidative stress but also inflammation [1]. In this regard, molecular docking studies have underlined that CUR and its analogs act as effective cyclooxygenase- (COX-) 2 inhibitors [58]. Moreover, they also inhibit the secretion of several inflammatory cytokines, such as chemokines, interleukins (ILs), and other inflammatory enzymes, including nitric oxide synthase, thereby attenuating the overall cytokine-associated proinflammatory environment and inhibiting the chronic ROS production [2, 6, 56, 57]. In addition, it has been stated that CUR exerts AIF actions through the suppression of nuclear factor-kappa B (NF-*κ*B) activity and stimulation of peroxisome proliferator-activated receptor-gamma pathway [59] and at the same time inhibiting a number of kinases, including protein kinase C [58]. In this regard, CUR can counteract inflammatory and oxidative processes by inhibiting NF-*κ*B activation (inhibiting IkB*α* kinase and AKT), which in turn suppresses the actions of all mediators connected with cell apoptosis, proliferation, invasion, and angiogenesis [60, 61]. CUR anti-inflammatory properties result from its modulatory action on several intracellular pathways, as highlighted by its ability to bind both COX-2 and 5-LOX and inhibiting their activity. [62]. Another important target of CUR's effectiveness is the Toll-like receptor- (TLR-) 4, which engagement activates important signals involved in the immune response modulation, as well as in cytokines and inflammatory chemokine production. INdde, scientific outcomes have shown that CUR mitigates inflammatory response through the direct action on TLR-4 or on its downstream route [42].

To cite some examples, in an *in vivo* study using the carrageenan-induced inflammatory test, cotreatment of *C. longa* and *Allium hookeri* Thwaites extracts resulted in effective suppression of inflammatory cytokine production and fast recovery of skin morphological changes in rats [5]. It has also been demonstrated that CUR has a crucial role in decreasing endometriosis progression by blocking oxidative stress, inflammation, and angiogenesis [63]. Similarly, turmeric extract supplementation inhibits inflammation and muscle damage in athletes [64], while nano-CUR supplementation exerts inflammation decreasing effects in females with metabolic syndrome [65]. CUR is also a potent inhibitor of the TLR-4-mediated action, which plays a significant role in the immune response regulation by improving inflammatory cytokine production [42]. CUR intraperitoneal injection (100 mg/kg) alleviates acute neuroinflammatory injury in mice via TLR4-mediated mechanism [66]. This polyphenol also modulates essential T-lymphocytes functions [11], indicating to be helpful in the treatment of T-helper-mediated inflammatory and autoimmune diseases [67]. CUR application (25 *μ*M) in glioma cells also triggered the reduction of critical inflammatory mediators such as the activator protein 1 (AP-1) and NF-*κ*B [68]. CUR can also downregulate the secretion of several proinflammatory cytokines such as TNF-*α*, IL-1, IL-6, and IL-12, thus modulating their target cell functions [68]. Via NF-*κ*B inhibition, CUR can also block the TNF-*α*-stimulated T-cell attachment to endothelial cells by reducing the expression of vascular cell adhesion molecules (VCAM)-1, intracellular adhesion molecules (ICAM)-1, and endothelial leukocyte adhesion molecules (ELAM)-1 [68].

Taken together, the reported data indicated CUR as a naturally occurring molecule that can effectively alleviate inflammation-associated diseases and their clinical manifestations compared to commonly used drugs [4, 69]. In a clinical study with anterior uveitis-baring patients, 2 weeks of CUR administration triggered a significant disease remission [4]. Also, it was proven that tolfenamic acid and CUR coadministration enhances tolfenamic acid AIF effects while reducing its toxic effects on the stomach and liver [70]. Moreover, CUR has been proposed to alleviate chronic inflammation after chemotherapy or radiotherapy [57]. A recent study revealed that CUR, vitamin C, and glycyrrhizic acid coadministration helps regulate immune response and fight the severe acute respiratory syndrome-coronavirus 2 (SARS-CoV-2) outcome by preventing the cytokine storm and inhibiting the plethoric inflammatory level [71]. In cases of ulcerative colitis-associated chronic colon inflammation, CUR has also been revealed to promote disease remission by acting in an NF-*κ*B-dependent fashion. Besides ameliorating inflammatory bowel disease, clinical trials have also proven CUR effectiveness in patients with gastric ulcers after 12 weeks of oral administration [4]. Dietary CUR consumption has been reported to attenuate myeloperoxidase (MPO) activity and leucocyte infiltration, simultaneously downregulating the levels of proinflammatory cytokines in intestinal diseases connected with oxidative stress and chronic inflammatory processes [72, 73]. In addition, CUR treatment has been also linked to reduced NO and O_2_^−^ levels along with the inhibition of NF-*κ*B activation in the colonic mucosa, confirming thus its beneficial effects in experimental colitis and valuable application in inflammatory bowel disease treatment.

Following CUR treatment, interesting achievements have been reported in rheumatoid arthritis (RA) [74]. RA is a chronic proinflammatory disease featured by uncontrolled synovial fibroblast growth where smoking and stress have been identified as leading causes [75, 76]. Most RA treatments so far in use are focused on pain and disability reduction, delay in disease progression, and improvement in patients' quality of life. Although AIF and antirheumatic drugs are the best choices for RA treatment [77], they are linked to undesired side effects, significantly reducing patients' compliance. In this sense, CUR has proven to be a valid adjuvant in treating arthritis [78]. Important immunomodulatory properties have been attributed to CUR [79]; among them is the ability to suppress the TNF-*α* expression in primary chondrocytes reducing cartilage breakdown [80] and inhibit inflammatory processes associated with arthritis [81].

By counteracting the release of essential inflammation mediators [82], CUR may also exert beneficial effects in several skin diseases including psoriasis [83]. In addition, CUR is able to inhibit keratinocyte proliferation, corroborating its effectiveness in psoriasis treatment [84, 85]. Psoriasis is a highly diffuse and painful disorder involving NF-*κ*B, signal transducer, and activator of transcription (STAT)-3 and TNF, which is usually treated with corticosteroids [86]. Nonetheless, as in other diseases, many of the most promising therapies are also linked to undesired side effects and often therapy failure; therefore, CUR can be a valuable and safe alternative. Moreover, CUR has shown significant wound healing properties [87], facilitating tissue remodeling, granulation tissue formation, and collagen deposition [88].

By interacting with redox-regulated copper/iron-bound proteins, CUR can inhibit oxidative stress preventing and counteracting the development of neurological disorders, such as AD and PD, and thus preventing cognitive impairment [89–91]. Furthermore, CUR also counteracts Alzheimer's disease- (AD-) associated damaging plaques and restore injured neurites in a mice model AD [92]. In addition, by suppressing the expression of TLR-4, high-mobility group box 1 protein, and receptor for advanced glycation, CUR can effectively inhibit microglial neuroinflammation in AD patients [93]. Through its antioxidant and AIF properties, CUR has also been shown to counteract atherosclerosis development, reduce myocardial ischemia and/or infarction damages, and prevent chemotherapy-induced cardiotoxicity in cancer patients [94–96].

Despite its numerous therapeutic benefits, physicochemical property-derived CUR poor bioavailability results in its poor absorption and rapid metabolism [97]. Moreover, its high liver metabolism and fast elimination also reduce CUR therapeutic effectiveness, especially in oral-administered formulations [98]. In this context, nanocarriers have appeared as a promising strategy to improve the CUR bioavailability and, consequently, its therapeutic effects. Indeed, thanks to their nanometric size and physicochemical property easy tuning; nanoparticles [99], liposomes [100–102], micelles, and phospholipid vesicles [103] are capable of potentiating CUR effectiveness at the desired level. In addition, as observed for several naturally occurring compounds, it is worthy of note that CUR antioxidant and AIF effects can be related to its metabolites, tetrahydro-CUR and octahydro-CUR [15, 104, 105]. In this regard, following tetrahydro-CUR and octahydro-CUR administration, a dose-dependent inflammation inhibition was observed in experimental mice models of acute inflammation. Noteworthy, tetrahydro-CUR and octahydro-CUR effect resulted more satisfying than CUR, especially in terms of COX-2 and NF-*κ*B pathway suppression [104].

## 4. Nanotechnology for Curcumin-Enhanced Efficacy

The achievement of controlled and targeted drug delivery has been studied for many years and appeared as a new challenge for pharmaceutical research, with nanomedicine emerging as a promising tool [106]. Nanotechnology has significantly changed the therapeutic perspective of several drugs, giving new and effective alternatives, especially for treating chronic diseases and many types of cancer [106]. Regardless of their structure and composition, and due to their small size and high surface area, nanosystems can modify the pharmacokinetic features of bioactive molecules, especially those characterized by low bioavailability rates. Nanosystems include nanoscale formulations or nanocarriers, which may ensure passive or active drug targeting, and improve drug circulation time and biodistribution, simultaneously protecting the incorporated drug from exogenous (i.e., light and heat) and endogenous (i.e., acid media, enzymes, and first-pass effect) degradation insults [107]. Moreover, nanotechnological carriers' research attention increased significantly in recent years as emerging nanosystems can coload therapeutic mediators and coordinate the delivery to specific target cells [108].

The promising findings obtained with nanocarrier-based drug delivery systems have raised researchers' interest in improving the efficacy of natural molecules, which are broadly conceived as safer and less expensive than synthetic drugs and effective for treating various diseases [109]. Combining folk medicine-derived biological molecules with new pharmaceutical nanotechnologies has emerged as a significant advance in developing new and safe therapeutic systems [110].

Given its excellent bioactive and therapeutic effects, CUR has aroused the scientific community's attention [22]. However, CUR yield extraction depending on the plant content in bioactive, besides being strongly affected by the extraction methodologies and solvents used [111]. In this regard, although several extraction methods have been reported, many of them are not suitable for industrial applications since they are energy-dissipative or involve toxic extraction solvents.

### 4.1. Curcumin Extraction Methods

CUR can be separated from *C. longa* rhizomes using different methods. Turmeric grinding and powder extraction has been performed since ancient times. Recently, additional and improved extraction methods have been explored and tested [33, 112, 113]. Among all, solvent extractions followed by column chromatography and Soxhlet, and ultrasonic and microwave extractions have become the most used because of the high reproducibility and extraction [114–116]. Also, a particular focus has been devoted to developing suitable and scalable extraction methods given the CUR rising demands from pharma, food, and cosmetic industries [33]. In this light, less expensive green extraction techniques capable of providing good yield have been chosen and accurately modified by researchers in order to be easily transferred at the industrial level [117]. As a result, pulse ultrasonic and microwave-assisted extraction methods, especially when high temperatures are employed, have been recently tested and chosen as innovative methods to improve CUR yield extraction [114, 118].

### 4.2. Curcumin-Loaded Nanocarriers

Pharmaceutical nanotechnology embraces intelligent and innovative systems or carriers characterized by nanometric size, which can be obtained using different materials, including polymers (either natural or synthetic), lipids, oils, surfactants, and other additives [119]. Main challenges that must be considered during nanocarrier formulation are payload physicochemical properties and biological barriers and defense mechanisms activated by the human body [120]. Among the different carriers tested for CUR delivery, phospholipid vesicles have largely been used and demonstrated to be the most effective in improving its stability and bioavailability irrespective of the chosen administration route [121–123]. The most used phospholipid vesicles are liposomes, mainly composed of phospholipids and water [124]. Due to their composition, they are highly biocompatible and can be appropriately modified with other additives such as water cosolvents, surfactants, lipids, polymers, and fibers to improve their delivery performance.

## 5. Curcumin Nanoformulations in Inflammatory Diseases

### 5.1. Curcumin-Loaded Nanocarriers in Pulmonary Ailments

It has been reported that CUR-loaded phospholipid vesicles can significantly improve CUR's anti-inflammatory properties, enhancing its overall therapeutic efficacy [99, 125]. In this light, new CUR-loaded phospholipid vesicle formulations and studies testing their potential in treating pulmonary disorders, such as asthma or chronic obstructive pulmonary diseases, have progressively increased. For instance, Manca et al. [126] formulated and used glycerosomes, which are vesicles containing high amounts of glycerol employed for CUR lung delivery through aerosol therapy. In this study, glycerosome formulation was improved by adding sodium hyaluronate or trimethyl chitosan chloride to ameliorate vesicle stability and performances during aerosolization process. The improved polymer-glycerosomes could deliver CUR in the last stages of the next-generation impinger to a better extent than regular glycerosomes. Moreover, glycerosomes in general and polymer-glycerosomes in particular, significantly improved CUR effectiveness by (i) inhibiting proinflammatory cytokine production (IL-6 and IL-8) and protects oxidatively stressed A549 cells in vitro and (ii) increasing CUR deposition in the deeper respiratory tract *vivo*. Similarly, Manconi et al. [127] formulated chitosan- and hyaluronan-coated liposomes for CUR pulmonary delivery and addressed carriers' influence on its effectiveness against oxidative stress. CUR incorporation in liposomes or polymer-coated liposomes significantly promoted CUR lung deposition and improved its antioxidant power, a phenomenon likely due to vesicles' ability to interact with cells and release CUR in the cytoplasm. CUR-loaded liposomes were also tested as an antiasthmatic system [128], leading to a significant reduction of inflammatory markers, such as IL-6, IL-8, IL-1*β*, and TNF-*α* compared to positive control. In this regard, the lower CUR-tested dosage (1 *μ*g/mL) reduced the inflammatory markers release to a better extent than higher doses, which is not surprising considering that natural compound-beneficial effects are now recognized to be influenced by several factors, including dose and redox environment [129–135]. Other studies also demonstrated that CUR liposomal formulations effectively reduced the expression of proinflammatory markers (IL-6, IL-8, and TNF-*α*) in human synovial fibroblasts and mouse macrophages (RAW264.7) stressed with LPS [136, 137]. Cytokine storm, which refers to the increased secretion of cytokines such as IL-1*β*, IL-6, TNF-*α*, and IL-18 is a characteristic of COVID-19 patients with lung damage [138, 139]. In this regard, a clinical trial performed on COVID-19 patients indicated the ability of a nanomicellar form of CUR to significantly decrease the mRNA expression and cytokine secretion levels of IL-6 and IL-1*β* [140], which may ameliorate disease's clinical manifestation and promote overall recovery.

### 5.2. Curcumin-Loaded Nanocarriers in Skin Ailments

Curcumin has also been incorporated in phospholipid vesicles tailored for skin applications. Hyalurosomes, a new class of phospholipid vesicles immobilized with sodium hyaluronate, have been specifically formulated to treat skin wound-associated inflammatory and oxidative processes [102]. Thanks to their peculiar structure and viscosity, hyalurosomes vesicles could incorporate a high CUR amount and retain it over 3 months of storage [102]. CUR-loaded hyalurosomes significantly improved CUR antioxidant activity being able to effectively protect keratinocytes from oxidative stress and even promoting cell proliferation [102]. Hyalurosomes also promoted CUR accumulation in different skin strata and wound healing *in vivo* in a mouse model of 12-O-tetradecanoylphorbol-13-acetate- (TPA-) induced lesions by inhibiting edema and MPO activity [102]. CUR-loaded phospholipid vesicles have also been used to reduce psoriasis-associated inflammatory and oxidative processes. In this regard, vesicles facilitated lipophilic payload penetration in different skin layers, ensuring its delivery in the damaged site [141, 142]. Recently, Zhang et al. [143] formulated hyaluronic acid-enriched ethosomes as topical systems for the treatment of psoriasis. In this work, hyaluronic acid was added to vesicle surface as it can interact with CD44 protein, which is overexpressed in inflammation- and oxidative stress-associated diseases and can be considered a potential targeting system capable of increasing both CUR skin retention and efficacy [144]. As expected, the CUR cumulative amount detected in the skin following hyaluronic acid-modified ethosome application was very high. This result may be due to the ethosomal bilayer's high flexibility that may overcome the stratum corneum barrier and reach the deepest skin strata, especially in the dermis, where psoriatic skin lesion-associated inflammatory cytokines, such as IL-17 and -22, are mainly located. *In vitro* results were confirmed by confocal observation of CUR accumulation in the skin, which was more evident in the deeper skin strata when hyaluronic acid-associated liposomes were used [144]. In particular, the CUR-associated fluorescence was preferentially located in the epidermis, where CD44 is highly expressed in psoriasis-like skin, thus promoting improved CUR accumulation at the inflammation site. Improved skin CUR accumulation is also linked to a keratinocyte's CUR significant uptake mainly because its incorporation into these flexible polymer-associated liposomes promotes “vesicle-cell” interaction and CUR internalization [144]. CUR-loaded chitosan nanoparticles linked with epidermal growth factor (EGF) were synthesized to develop an EGF-modified spray solution (EGF@CCN) for treating skin wounds [145] Such nanoformulation tested in a Wistar rats' model of full-thickness dermal defect shows the ability to promote an almost complete wound healing after 12 days postoperation [145].

CUR-alginate-based nanomicelle (C-A-NM) also show colonic wound healing properties in rats as evidenced by both histopathology/colonoscopy evaluation and increased protein and collagen synthesis in damaged sites [146]. C-A-NM also increased TGF*β*1 expression while decreasing that of NF*κ*B, a phenome that may explain the observed healing effect [146]. A randomized clinical trial performed with different CUR nanoformulation showed that CUR-loaded nanostructured lipid carriers (NLC) are able to provide a better drug delivery and physiological skin parameters ameliorations as compared to nanosized emulsions based on monoacyl-phosphatidylcholine (MAPL) [147]. Further clinical studies are needed to better understand the optimal nanoformulation able to provide the best therapeutic results in the different pathological conditions.

### 5.3. Curcumin-Loaded Nanocarriers in Rheumatoid and Osteoarthritis

CUR-loaded hyalurosomes were also tested as topical carriers for the treatment of RA [148]. Briefly, RA is a chronic inflammation due to an uncontrolled proliferation of fibroblast-like synovial cells responsible for the release of proinflammatory cytokines [149]. Treatment of fibroblast-like synovial cells with CUR-loaded hyalurosomes significantly inhibited IL-15 and IL-6 production, key molecules in RA pathogenesis. Moreover, CUR-loaded hyalurosomes stimulated the production of IL-10, which is considered the most important AIF cytokine. CUR-loaded hyalurosomes also suppressed NF-*κ*B release and ROS production, confirming the double effect of this CUR formulation in reduction of joint damage by inhibition of TNF-*α* and ROS generation [148].

CUR-loaded liposomes enriched with cholesterol have been formulated for the treatment of osteoarthritis. Data indicated that this new CUR nanosystem formulation provided improved 7F2 osteoblastic cell survival and bioactive accumulations into cells [150]. They were also capable of inhibiting NO production in stimulated RAW264.7 macrophages and preventing osteoclast differentiation by downregulating cathepsin K and tartrate-resistant acid phosphatase (TRAP) expression. Moreover, reduced levels of IL-1*β*-induced COX-2 and MMP-3 were also detected in 7F2 osteoblasts exposed to these CUR-loaded cholesterol-enriched liposomes. Therefore, CUR-loaded liposomes may have a promising effect against subchondral bone turnover slowing the osteoarthritis progression [150]. In this regard, a recent clinical trial reports that a nanomicellar formulation of CUR significantly improves the symptoms of osteoarthritis patients [151]. Indeed, an overall improvement of pain, stiffness, and physical activity subscales of the Western Ontario and McMaster Universities Osteoarthritis Index (WOMAC) questionnaire were found in treated patients compared with the placebo group [151].

### 5.4. Curcumin-Loaded Nanocarriers in Neurodegenerative Disorders

The antioxidant and AIF potential of CUR-loaded nanosystems has also been proven in the treatment of neurodegenerative disorders [152]. Wheat germ agglutinin-conjugated liposomes incorporated with cardiolipin reduced SK-N-MC cell neurodegeneration and amyloid-*β* plaque deposition providing neuronal protection in a rodent model of AD [153]. In another study, Sokolik [154] demonstrated that CUR-loaded liposomes reduced angiotensin-converting enzyme activity in brain-targeted regions potentiating memory recovery in rats with AD. A set of clinical trial provided evidence regarding the ability of *ω*-3 fatty acids and nanocurcumin combination to significantly reduce migraine attack frequency [155–158]. The drug combination was able to reduce the serum levels of VCAM and C-reactive protein (CRP) along with proinflammatory cytokines IL-1*β* and IL-6, which may be the mechanism at the basis of the reported therapeutic effect [155–158].

### 5.5. Curcumin-Loaded Nanocarriers in Cardiovascular Diseases

**Figure 3:**
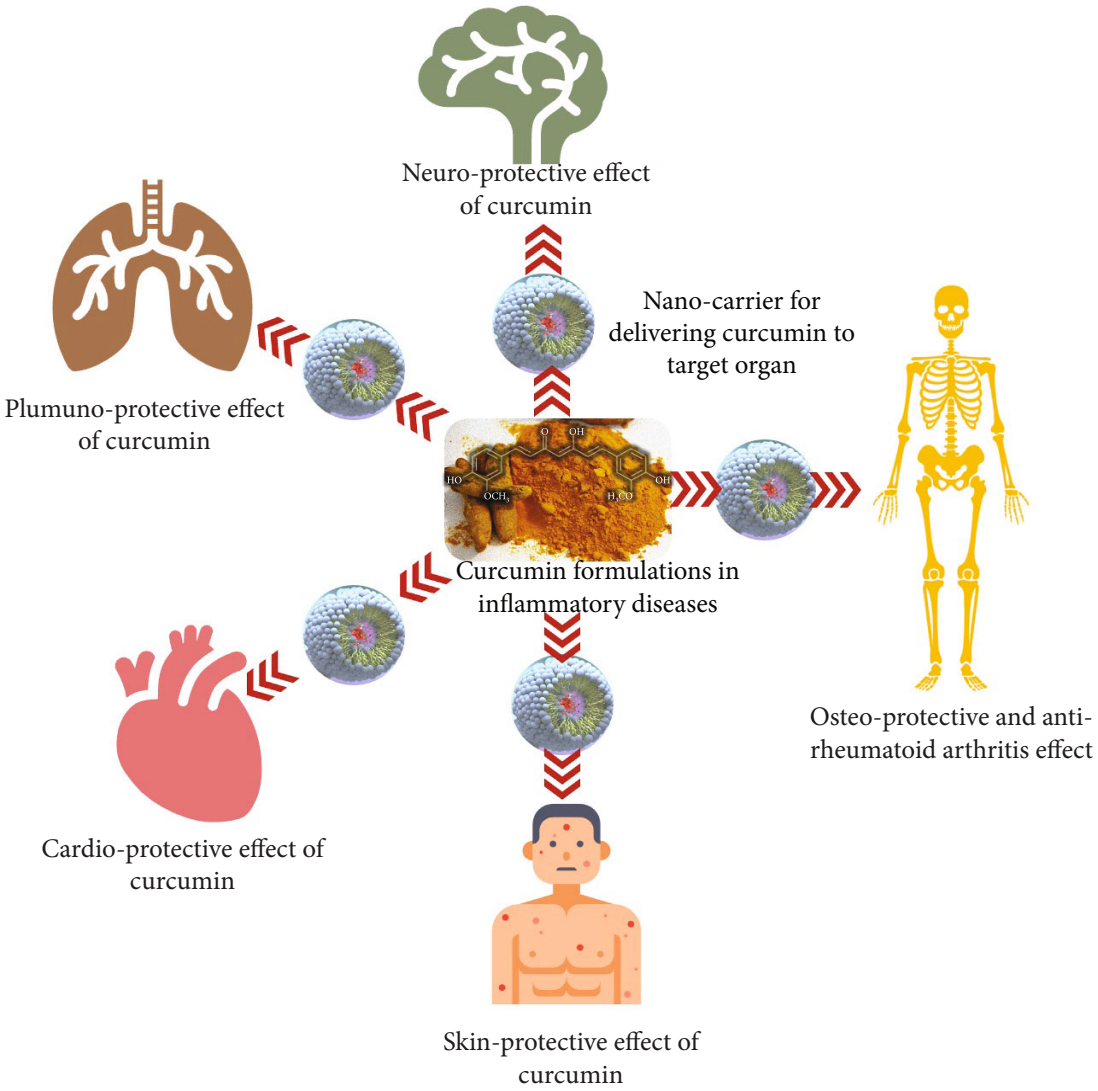
Protective effect of curcumin-loaded nanocarriers on various ailments in human body.

CUR-loaded phospholipid vesicles have also been proven to be effective in the treatment of CV diseases. It is now widely accepted that CV diseases such as hypertension and atherosclerosis result from endothelial cell dysfunction induced by inflammatory factors and plasma lipid deposition at the damaged sites [159, 160]. Liposomes specifically designed to codeliver atorvastatin calcium and CUR for atherosclerosis treatment have been recently proposed [161]. Atorvastatin calcium is currently used as an antiatherosclerotic drug, and CUR is a safe adjuvant capable of promoting antiatherosclerotic effects and reduces atorvastatin calcium cytotoxicity. Liposomes were surface modified with specific ligands capable of targeting vesicles at the desired level to improve payload antiatherosclerotic and AIF effects. Indeed, the combination of the two active substances effectively reduced atherosclerotic areas and proinflammatory factor levels [161]. Moreover, the vesicle surface functionalization further improved both drugs' effectiveness suggesting their possible use for preventing or treating endothelial cell disfunction-associated CV diseases. Consonant with the above-reported findings, the AIF, antioxidant, and CV protective effects of CUR-loaded liposomes have been previously reported [29, 125]. A recent clinical trial reports that CUR nanomicelle (80 mg/day) ameliorates lipid profile and oxidative and inflammatory markers in patients undergoing coronary angioplasty [162]. The same trial indicates that the CUR nanoformulation provides significantly better result in lowering triacylglycerol (TG), total cholesterol (TC), malondialdehyde (MDA), superoxide dismutase (SOD), and tumor necrosis factor-alpha (TNF-*α*) levels as compared to CUR alone (500 mg/day) [162]. Another trial performed in hemodialysis patients shows that nanocurcumin (120 mg/day) decreases the serum levels of CRP, along with VCAM-1 and ICAM-1, two proinflammatory adhesion molecules involved in endothelial dysfunction [163]. CUR nanomicelle also significantly improve the levels of TG in patients with metabolic syndrome, while failed to ameliorate other biochemical parameters such as TC, LDL-C, HDL-C, fasting blood sugar (FBS), hemoglobin A1c (HbA1c), homeostatic model assessment (HOMA) for insulin resistance (HOMA-IR), and pancreatic *β* cell function (HOMA-*β*) [164]. The various protective effects of CUR-loaded nanocarriers on different human body ailments are illustrated in Figure 3.

## 6. Role of Nanocarriers in Curcumin Bioavailability and Stability

Due to its low absorption and degradation at gastric and liver levels, the lowest bioavailability of CUR is generally obtained following oral administration [165]. However, since oral administration is the patients' preferred route of administration, many efforts have been carried out to find effective oral formulations of CUR, especially using nanocarriers. For this purpose, CUR has been loaded in phospholipid vesicles enriched with fibers and polymers called nutriosomes [166]. Nutriosomes resulted stable in acidic and neutral environments mimicking the gastrointestinal tract and improved the protective activity of CUR against hydrogen peroxide-stressed CaCo2 cells. These findings appear mainly connected with an enhanced “nutriosome-cell” interaction confirmed by the improved vesicle internalization detected by confocal microscopy. Nutriosomes increased the *in vivo* CUR biodistribution and reduced the 2,4,6-trinitrobenzene sulfonic acid- (TNBS-) induced intestinal inflammation in rats [167].

In a similar study, CUR was incorporated into eudragit-hyaluronan multicompartment liposomes aiming at improving its intestinal bioavailability [100]. The long-term vesicle stability was ensured by their lyophilization. However, they can be easily rehydrated in liquid or semisolid foods to obtain an extemporaneous CUR-enriched food with beneficial properties for human health [100]. Due to their multicompartmented structure, these vesicles have shown remarkable stability under the gastrointestinal tract harsh conditions, such as high ionic strength and pH variations, and ensured a more significant CUR deposition in the intestines in comparison with CUR dispersion. Indeed, CUR-containing vesicles could reach intact intestines to exert the potential therapeutic effect by releasing their payload [100]. The use of polymers as a phospholipid vesicle stabilizer seems promising for formulating delivery systems specifically designed for oral administration. In this regard, De Leo et al. [168] prepared Eudragit S100-coated liposomes with the intent to improve vesicle stability. The coating process allowed a polymer layer formation, which efficiently protected both the liposomes and the incorporated CUR. Indeed, CUR released experiments have proved the polymeric layer high stability and ability to protect CUR against degradation after treatment of coated liposomes with bile salts capable of destroying liposomes [168]. Moreover, eudragit-coated liposomes also preserved CUR antioxidant property under the gastrointestinal tract's harsh conditions as its antioxidant activity was detected only when the layer was dissolved in basic pH condition mimicking the intestinal environment.

## 7. Conclusions and Future Perspective

This review's finding highlights the CUR potential as a natural and safe bioactive for treating different inflammation- and oxidative stress-related diseases. Nonetheless, numerous studies highlighted the CUR limitations when employed as a free drug because of its low bioavailability and regardless of the administration route chosen. In this light, studies developing and investigating CUR nanoformulations as a way to improve its bioavailability and effectiveness are exponentially increasing. In this regard, liposomes and derived phospholipid vesicles disclosed promising performance in CUR delivery to the lungs, gut, and skin. Overall, data obtained in the last years confirmed the potential of phospholipid vesicles as ideal carriers for CUR, especially when they are ad hoc formulated for a specific route of administration and a particular disorder by using appropriate additives or ligands. Noteworthy, most of the available literature reports in vitro or animal models data while in vivo clinical studies are so far scarce. Nonetheless, some of the available clinical trials provide the first evidences that CUR nanoformulations may wield better result in terms of therapeutic effect as compared to CUR alone. Moreover, nanocombinations of CUR with other natural compounds or currently used drugs appear another promising way to improve the overall therapeutic effect of the employed compounds. In this regard, more research effort should be directed at understanding whether CUR nanoformulations may be routinely included into the standardized therapeutic treatments to reduce the amount of the main drugs with the intent to obtain improved therapeutic effect with less toxicity. Moreover, simple, cost-effective and scalable technologies to produce effective CUR nanocarrier should also be developed in order to make the “nanodrug sector” competitive in the pharmaceutical industry towards the classic drugs. As evidenced by this review, to better maximize CUR efficacy, different and specific nanoformulations are required, and larger and deeper clinical studies should be carried out to understand its pharmacokinetic behavior and *in vivo* therapeutic effects.

## Data Availability

The data supporting this review are from previously reported studies and datasets, which have been cited. The processed data are available from the corresponding author upon request.
